# Supplementary material for: Evaluation of Biologics ACE2/Ang(1–7) Encapsulated in Plant Cells for FDA Approval: Safety and Toxicology Studies
Source: Pharmaceutics. 2024 Dec 25;17(1):12. doi: 10.3390/pharmaceutics17010012 (PMC11768411; doi:10.3390/pharmaceutics17010012)
Supplement: Supplementary file 1 [file pharmaceutics-17-00012-s001.zip › Figure S2 Summary of body weight gains.pdf]

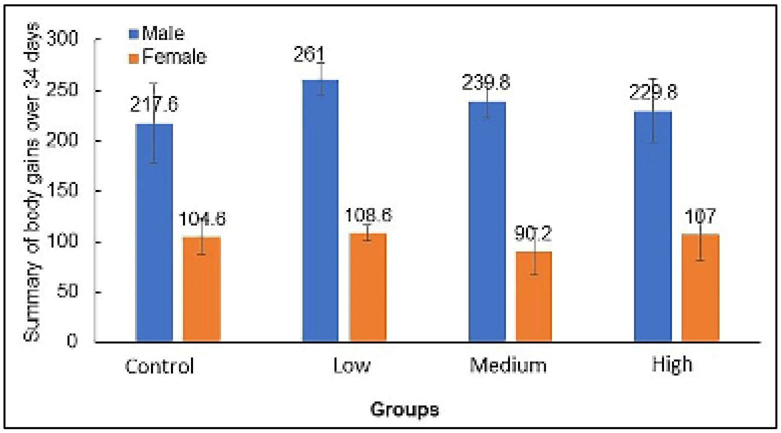

Figure S2: Summary of body weight gains. Data represents the body gains (g) over 34 days relative to start date. Data represented as mean ± SD (n= 5). ANOVA with Dunnett's method showed that the low, medium and high doses did not produce significant difference in the body weight gains than that of placebo control.
