# Supplementary material for: Evaluation of Biologics ACE2/Ang(1–7) Encapsulated in Plant Cells for FDA Approval: Safety and Toxicology Studies
Source: Pharmaceutics. 2024 Dec 25;17(1):12. doi: 10.3390/pharmaceutics17010012 (PMC11768411; doi:10.3390/pharmaceutics17010012)
Supplement: Supplementary file 1 [file pharmaceutics-17-00012-s001.zip › Figure S3 Summary of food consumption.pdf]

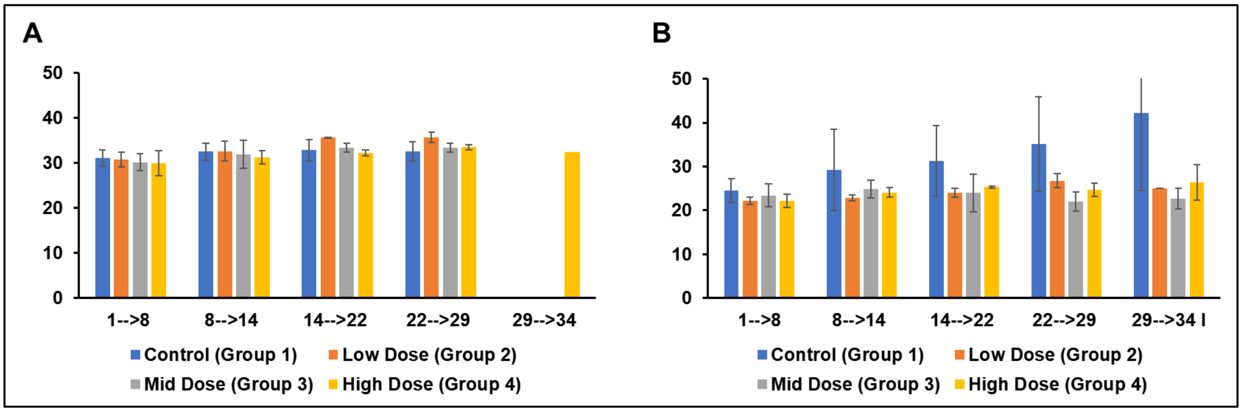

Figure S3: Summary of food consumption. Food Mean Daily Consumption (g/animal/day) over 34 days relative to start date. **(A)** Food consumption (g/animal/day) by male rats. **(B)** Food consumption (g/animal/day) by female rats. Data represented as mean ± SD (n=5). ANOVA with Dunnett's method showed that the low, medium and high doses did not produce significant difference in test parameters than that of placebo control.
