# Supplementary material for: Evaluation of Biologics ACE2/Ang(1–7) Encapsulated in Plant Cells for FDA Approval: Safety and Toxicology Studies
Source: Pharmaceutics. 2024 Dec 25;17(1):12. doi: 10.3390/pharmaceutics17010012 (PMC11768411; doi:10.3390/pharmaceutics17010012)
Supplement: Supplementary file 1 [file pharmaceutics-17-00012-s001.zip › Table S1A Formulation of investigational product.pdf]

Table – S1A: Formulation of investigational product CTB-ACE2 and CTB-Ang (1-7) doses for oral gavage.

Gr. No.	Test Material	Dose (mg IP/kg/dose)	Dose (mg IP/kg/day)[a]	mg Plant powder/kg/dose)[b]	Total mg Plant powder/kg/dose	Dose Volume[c] (mL/kg)	Dose Conc. (mg/mL)	No. of Animals			
								Main Study		Recovery Study	
								M	F	M	F
1	Placebo[d]	0	0	460.13	460.13	10	46.0	10	10	5	5
2	ACE2	0.8	1.6	109.29	460.13	10	46.0	10	10	5	5
	Ang (1-7)	0.5	1.0	120.77							
	Placebo	0	0	230.07							
3	ACE2	1.6	3.2	218.58	460.13	10	46.0	10	10	5	5
	Ang (1-7)	1.0	2.0	241.55							
4	ACE2	4.16	8.3	568.31	1196.3	20	59.8	10	10	5	5
	Ang (1-7)	2.5	5	628.02							

Gr=Group; No.=Number; IP=Investigational Product: recombinant ACE2/Ang (1-7); Conc.=Concentration; M=Male; F=Female.

[a] Dosed via oral gavage twice daily for 14 days.

[b] Assumes 7.32 mgACE2 and 4.14 mg Ang (1-7) per 1gram lettuce plant powder.

[c] Based on the most recent body weight measurement.

[d] Wildtype biomass.
