# Supplementary material for: Evaluation of Biologics ACE2/Ang(1–7) Encapsulated in Plant Cells for FDA Approval: Safety and Toxicology Studies
Source: Pharmaceutics. 2024 Dec 25;17(1):12. doi: 10.3390/pharmaceutics17010012 (PMC11768411; doi:10.3390/pharmaceutics17010012)
Supplement: Supplementary file 1 [file pharmaceutics-17-00012-s001.zip › Table S1B Toxicokinetic study design.pdf]

Table – S1B Toxicokinetic study design

Gr. No.	Test Material	Dose Level (mg IP/kg/dose)	Dose Level (mg IP/kg/day)	Plant powder (mg/ kg/dose)[a]	Total mg Plant powder/kg/dose	Dose Volume[b] (mL/kg)	Dose Conc. (mg/mL)	No. of Animals Toxicokinetic Study	
								Males	Females
1	Placebo	0	0	460.13	460.13	10	46	3	3
2	ACE2	0.8	1.6	109.29	460.13	10	46	6	6
	Ang (1-7)	0.5	1	120.77					
	Placebo	0	0	230.07					
3	ACE2	1.6	3.2	218.58	460.13	10	46	6	6
	Ang (1-7)	1	2	241.55					
4	ACE2	4.16	8.3	568.31	1196.33	20	59.8	6	6
	Ang (1-7)	2.5	5	628.02					

Gr = group; No. = number; IP = investigational product: recombinant ACE2 or Ang (1-7); Conc. = concentration.

[a] Assumes 7.32 mg ACE2 per 1-gram ACE2 lettuce powder and 4.14 mg Ang (1-7) per gram of Ang (1-7) lettuce powder.

[b]Based on the most recent body weight measurement.
