# Supplementary material for: Evaluation of Biologics ACE2/Ang(1–7) Encapsulated in Plant Cells for FDA Approval: Safety and Toxicology Studies
Source: Pharmaceutics. 2024 Dec 25;17(1):12. doi: 10.3390/pharmaceutics17010012 (PMC11768411; doi:10.3390/pharmaceutics17010012)
Supplement: Supplementary file 1 [file pharmaceutics-17-00012-s001.zip › Table S3 Blood sample collection and processing for clinical pathology studies.pdf]

Table S3: Blood sample collection and processing for clinical pathology studies.

Group Nos.	Hematology	Coagulation	Clinical Chemistry	Urinalysis
1 to 4 – Blood samples from animals scheduled for euthanasia on Day 15	X	X	X	X
1 to 4 - Recovery study animals only on Day 35	X	X	X	X
Unscheduled euthanasia (when possible)	X	X	X	-
Fasting – Scheduled collection only	-	-	Min. 4 hours	Overnight
Method/Comments:	Venipuncture of the Vena Cava[a]	Venipuncture of the Vena Cava[a]	Venipuncture of the Vena Cava[a]	Urine will be collected by urine collection cages containing an alternate water source
Target Volume (mL)[b]:	0.5	1.8	2	-
Anticoagulant:	(K2) EDTA	Sodium citrate	None	-
Special Requirements:	-	-	-	-
Processing	None	Plasma	Serum	-

X = Sample to be collected; Min. = Minimum; - = Not applicable.

[a] Was collected under isoflurane anesthesia. On days of unscheduled euthanasia, blood may be collected under isoflurane anesthesia from the jugular or tail vein.

[b] Additional samples obtained (e.g., due to clotting of non-serum samples) if permissible sampling frequency and volume are not exceeded.
