# Supplementary material for: Evaluation of Biologics ACE2/Ang(1–7) Encapsulated in Plant Cells for FDA Approval: Safety and Toxicology Studies
Source: Pharmaceutics. 2024 Dec 25;17(1):12. doi: 10.3390/pharmaceutics17010012 (PMC11768411; doi:10.3390/pharmaceutics17010012)
Supplement: Supplementary file 1 [file pharmaceutics-17-00012-s001.zip › Table S4 Computerized systems for data analysis.pdf]

Table S4: Computerized systems for data analysis of different toxicological parameters at Charles River Laboratories.

System Name	Version No.	Description of Data Collected and/or analyzed
Provantis®	10	In-life (clinical observations, body weights, food consumption, ophthalmology); clinical pathology (hematology, coagulation, clinical chemistry, urinalysis); postmortem (organ weights, macroscopic and microscopic examinations)
Systems 600 Apogee Insight System	3.15	Temperature and/or humidity (animal rooms, refrigerators, freezers, and compound storage, as applicable)
Instem Life Science Systems, DISPENSE	10	Test material receipt, accountability, and/or formulation activities
Advia 2120i with Multispecies System Software	6.3.2-MS	Hematology data
Cobas 6000 c501	06-03	Clinical chemistry data
Stago STA Compact Analyzer	108.06	Coagulation data
Clinitek Advantus Urine Dipstick Analyzer	V03.11:071/V3.10	Urinalysis data
Deviation Information Library	2.1	Deviations/Notes to File
M-Files	21.1	Reporting and collection of 21 CFR Part 11 compliant signature
Share Document Management System	1.0	Reporting
