# Supplementary material for: Evaluation of Biologics ACE2/Ang(1–7) Encapsulated in Plant Cells for FDA Approval: Safety and Toxicology Studies
Source: Pharmaceutics. 2024 Dec 25;17(1):12. doi: 10.3390/pharmaceutics17010012 (PMC11768411; doi:10.3390/pharmaceutics17010012)
Supplement: Supplementary file 1 [file pharmaceutics-17-00012-s001.zip › Table S2 In life assessment of parameters and frequency.pdf]

Table S2: In-life assessment of parameters and frequency.

Parameter	Population(s)	Frequency (minimum required)	Comments
Mortality	All Main, Recovery, and Toxicokinetic Study animals	At least twice daily (morning and afternoon) beginning upon arrival through termination/release.	Animals were observed within their cage unless necessary for identification or confirmation of possible findings.
Cage Side Observations	All Main and Recovery Study animals	At least once daily, from at least Week -1 and throughout the study. Cage side observations are not required on the days of detailed clinical observations during the pretreatment (prior to Day 1) and recovery periods, when a post dose observation is recorded, or on the day of scheduled euthanasia.	Animals were observed within their cage unless necessary for identification or confirmation of possible findings.
Post dose Observations	All Main and Recovery Study animals	At least once daily during the dosing period; 2 to 4 hours post the first daily dose.	Animals were observed within their cage unless necessary for identification or confirmation of possible findings.
Detailed Clinical Observations	All Main and Recovery Study animals	At least once weekly, from at least Week -1 and throughout the study.	Animals were removed from the cage.
Individual Body Weights	All Main, Recovery, and Toxicokinetic Study animals	At least once weekly, from at least Week -1 and throughout the study.	A fasted weight was recorded for main and recovery study animals on the day of scheduled euthanasia. Not collected from animals found dead or euthanized moribund.
Food Consumption	All Main and Recovery Study animals	Weekly; from at least Day 1 and throughout the study.	Quantitatively measured, except for on the day of scheduled euthanasia.
