# Supplementary material for: Evaluation of Biologics ACE2/Ang(1–7) Encapsulated in Plant Cells for FDA Approval: Safety and Toxicology Studies
Source: Pharmaceutics. 2024 Dec 25;17(1):12. doi: 10.3390/pharmaceutics17010012 (PMC11768411; doi:10.3390/pharmaceutics17010012)
Supplement: Supplementary file 1 [file pharmaceutics-17-00012-s001.zip › Table S5 Clinical observations.pdf]

Table S5: Clinical observations during the pretreatment (prior to Day 1) and recovery periods.

**Observation**	**Number of Animals Affected**							
	**Male**				**Female**			
Respiratory Rate Abnormal	Placebo Group 1	Low Group 2	Medium Group 3	High Group 4	Placebo Group 1	Low Group 2	Medium Group 3	High Group 4
Breathing, Labored	0	0	0	0	0	0	0	0
Breathing, Abnormal Sounds	0	0	0	0	0	0	1	0
Hunched Posture	0	0	0	0	0	0	0	0
Fur, Erected	0	0	0	0	0	0	0	0
Fur, Staining, Muzzle	0	0	0	0	0	0	0	0
Activity Decreased	0	0	0	0	0	0	0	0
